# Cyclopentadienyl Complexes of Technetium

**DOI:** 10.3390/molecules30244813

**Published:** 2025-12-18

**Authors:** Ulrich Abram, Maximilian Roca Jungfer

**Affiliations:** 1 Institute of Chemistry and Biochemistry, Freie Universität Berlin, Fabeckstr. 34/36, 14195 Berlin, Germany; 2 Institute for Nuclear Waste Disposal (INE), Karlsruhe Institute of Technology, Hermann-von-Helmholtz-Platz 1, 76344 Eggenstein-Leopoldshafen, Germany

**Keywords:** technetium, cyclopentadienyl, sandwich complexes, half-sandwich complexes, nitrosyl, carbonyl, nuclear medicine

## Abstract

The number of structurally investigated cyclopentadienyl (Cp^−^) complexes of technetium is limited in contrast to the situation with its heavier homolog, rhenium. Although this could be attributed to the radioactivity of all isotopes of the radioelement, there are also clear chemical differences to analogous compounds of the other group seven elements, manganese and rhenium. Technetium Cp^−^ compounds are known with the metal in the oxidation states “+1” to “+7”, with a clear dominance of Tc(I) carbonyls and nitrosyls. Corresponding carbonyl complexes also play a significant role in the development of ^99m^Tc-based radiopharmaceuticals with the aromatic ring as an ideal position for the attachment of biomarkers. In this paper, the present status of the synthetic and structural chemistry of technetium with Cp^−^ ligands is discussed, together with recent developments in the corresponding ^99m^Tc labeling chemistry.

## 1. Introduction

Element number 43, technetium, is extraordinary from different points of view. (i) It is the first chemical element that has been discovered from a man-made nuclear reaction before its existence in natural resources on earth could be reliably proven [1,2,3,4,5], (ii) it is the lightest element of the Periodic Table, which has exclusively radioactive isotopes [6], and (iii) despite, these two facts, it has gained considerable importance in technological processes [7] as the main component in numerous radiopharmaceutical procedures [8,9,10,11,12,13,14,15,16] and as a potential long term problem in the nuclear waste treatment [17,18,19,20]. This is almost exclusively due to two out of the more than fifty hitherto known technetium nuclides (38 isotopes + several nuclear isomers [6]): the long-lived ^99^Tc and its short-lived γ-emitting nuclear isomer ^99m^Tc.

The weak beta emitter ^99^Tc (half-life T_1/2_ = 2.11 × 10^5^ years, E_β,max_ = 297.5 keV) is the only technetium isotope that is available in macroscopic amounts from uranium fission. It is formed by the disintegration of ^99^Mo, which is approximately 6% of the main fission products in nuclear reactors. The relatively short half-life of ^99^Mo (T_1/2_ = 65.9 h) is the reason that the molybdenum converts practically completely into ^99^Tc within a few weeks. Thus, the worldwide amount of technetium produced by nuclear power plants increases annually by almost 10 tons [21]. This means that the current total global inventory of technetium amounts to several hundred tons. Fortunately, most of this technetium is still contained in spent fuel rods or in corresponding waste tanks. Since ^99^Tc causes serious problems in the reprocessing of nuclear fuel, it must be removed from spent fuel solutions before re-enrichment of 235-uranium. Consequently, sufficient quantities of the artificial element are available for chemical investigations, and all structural studies discussed in this work were carried out exclusively with this isotope. The long half-life and the low beta energy of ^99^Tc allow the handling of milligram amounts of this isotope without considerable hazards, provided that fundamental radiation protection regulations are respected. Normal glassware gives adequate protection against the weak beta radiation and bremsstrahlung only becomes relevant when larger amounts of ^99^Tc are handled. This allows the isolation of technetium compounds in crystalline form and the recording of spectroscopic and crystal structure data.

The metastable nuclear isomer ^99m^Tc (T_1/2_ = 6.01 h, E_γ_ = 142.7 keV) is formed in considerable amounts (approximately 90%) as an intermediate product during the ^99^Mo decay and finally disintegrates to the ground-state nuclide ^99^Tc. It emits almost pure γ-radiation in an energy range that is perfectly suitable for diagnostic applications in nuclear medicine. For this reason, ^99^Mo/^99m^Tc generator systems have been developed for application in nuclear medical clinics. They contain highly purified ^99^MoO_4_^2−^ tightly adsorbed, e.g., on a column of silica. The continuously formed disintegration product ^99m^TcO_4_^−^ can be eluted from this column by saline and used for the preparation of radiopharmaceuticals for the imaging of various organ systems. Despite the development of modern radiopharmaceuticals on the basis of the positron emitters ^18^F, ^11^C, or ^68^Ga, ^99m^Tc remains the workhorse in this field, with approximately 80% of the conducted clinical studies, which gives a number of ~40 million annual administrations worldwide [13,14,15,22].

Interestingly, organotechnetium compounds play a role in both radiopharmacy and nuclear waste management. For example, ^99^Tc carbonyl complexes are formed during the storage of technetium-containing nuclear waste in highly radioactive solutions such as the Hanford wastewater tanks, while also serving as key motifs for existing or prospective ^99m^Tc-based diagnostics in nuclear medicine. The structural chemistry of technetium with cyclopentadienyl (Cp^−^) ligands is relatively unexplored. In contrast to the situation with the other two Group 7 elements, manganese and rhenium, where more than 1000 compounds each have been investigated by crystal structure analysis, the number of well-known ^99^Tc cyclopentadienyls is small. Several new examples have been reported during recent years. The same holds true for ^99m^Tc cyclopentadienyl complexes, which have recently been developed as promising candidates for the coupling of radioactive technetium atoms to biologically active molecules.

Several excellent reviews covering general aspects of technetium chemistry [9,16,23,24,25,26,27], its environmental chemistry [17,28,29,30], the chemistry of organotechnetium compounds [12,31,32,33,34,35,36,37], and applications in nuclear medicine [8,9,10,11,12,13,14,15,16] have been compiled in the past. Thus, the present work focuses on cyclopentadienyl compounds of technetium and the related structural chemistry of ^99^Tc complexes, but also gives a summary of the recent progress with novel ^99m^Tc derivatives and their medicinal potential.

## 2. ‘Classical’ Cyclopentadienyl Chemistry

**Scheme 1:**
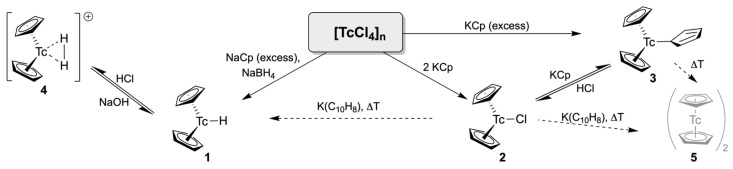
Syntheses and reactions of ‘classical’ technetium(III) Cp^−^ complexes.

The first cyclopentadienyl compounds of technetium were prepared in the early 1960s by the pioneers of organotechnetium chemistry [38,39,40] (Scheme 1). Subsequent reactions of the polymeric [TcCl_4_]_n_ with NaCp and NaBH_4_ gave the Tc(III) hydride [Tc(Cp)_2_H] (**1**) as a golden yellow, crystalline substance. Since no hydride resonance could be detected in the proton NMR spectrum of the diamagnetic product with the available 50 MHz CW NMR spectrometer, the dimeric structure [Tc(Cp)]_2_ was tentatively assigned [38]. The questionable signal was later detected as a broad (halfwidth: 40 Hz) signal at 17.8 ppm [39]. This halfwidth is indeed much larger than that of the signal in the analogous rhenium compound [Re(Cp)_2_H] (halfwidth: 6 Hz) [39]. Two possible reasons for the observed problem should be briefly mentioned. Significant line-broadenings are not unusual for NMR signals of atoms, which are directly bonded to technetium. They are frequently found in ^31^P or ^1^H NMR spectra of diamagnetic phosphine or hydrido complexes of technetium, particularly when the local symmetry of the ^99^Tc nucleus is low. Such interactions can cause extreme line-broadenings of the related ^31^P or hydrido resonances, which make their resolution even with modern NMR instruments frequently complicated [41,42,43]. A common explanation is given by scalar couplings with the large quadrupole moment of ^99^T (I = 9/2, Q = −0.19 × 10^−28^ m^2^) [44,45]. Another effect that can possibly contribute to the observed broadening of the resonance of the hydride ligand when going from the known rhenium compounds to their technetium analogs, might be the dynamic exchange effects due to the weaker M–H bonds in the technetium compounds, as has already been concluded by Fischer and Schmidt on the basis of the relative intensities of the [M(Cp)_2_H]^+^ and [M(Cp)_2_]^+^ (M = Tc, Re) peaks in the electron impact mass spectra of the respective hydrido compounds [38]. More detailed mass spectrometric studies confirmed this assumption by the estimation of the related M–H binding energies of 0.73 eV ([Tc(Cp)_2_H]) and 1.09 eV ([Re(Cp)_2_H]) [46].

Hydride **1** is a typical base and reacts with HCl under formation of the cationic dihydride (or better dihydrogen complex) **4** [38,39]. Such reactions are reversible, and the addition of NaOH results in the re-formation of monohydride **1**.

The Tc(III) chlorido complex [Tc(Cp)_2_Cl] (**2**) is obtained as a red-brown solid by a prolonged heating of (TcCl_4_)_n_ with two equivalents of KCp in THF, while a similar reaction with an excess of KCp or the treatment of **2** with one equivalent of KCp yield the red tris complex [Tc(Cp)_2_(η^1^-Cp)] (**3**) with one σ-bonded cyclopentadienyl ligand (Scheme 1) [47,48]. Both compounds have been characterized by X-ray diffraction, confirming that the bent coordination of the η^5^-bonded cyclopentadienyl rings together with a σ-bonded co-ligand. The treatment of compound **2** with potassium naphtalenide results in the formation of two new products: the golden-yellow hydrido complex **1** and a second, amber-colored compound, for which the dimeric structure **5** has been proposed on the basis of mass spectrometry. Attempts to substitute the Cl^−^ ligand in **2** by a methyl group during a reaction with methyllithium gave an oily, green product (probably [Tc(Cp)_2_Me] [47]). The σ-bonded Cp^−^ ligand in the tris-complex **3** is labile and can be replaced by chloride during reactions with HCl or NH_4_Cl [47].

**Figure 1:**
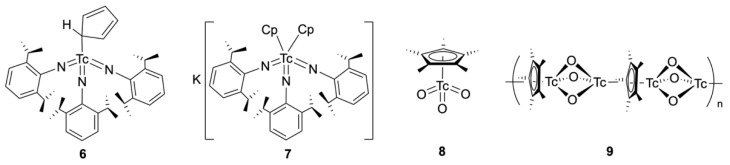
High-valent Cp^−^ complexes of technetium.

A monodentate, σ-bonded Cp^−^ ligand is also found in the technetium(VII) tris-imido complex [Tc(η^1^-Cp)(NAr)_3_] (Ar = 2,6-diisopropylphenyl) (**6**), shown in Figure 1. It has been obtained by the reaction of the corresponding iodido complex [TcI(NAr)_3_] with one equivalent of KCp in THF [49] and represents a structural analog of the corresponding methyl derivative [MeTc(NAr)_3_]. This has been prepared in a similar reaction with MeLi [50]. The use of two equivalents of KCp in the reaction with [TcI(NAr)_3_] yields the potassium salt K[Tc(Cp)_2_(NAr)_3_] (**7**) as a blue solid. The compound has been studied by ^1^H and^13^C NMR spectroscopy and its composition has been proven by an elemental analysis on the bis(triphenylphosphine)iminium salt. Spectroscopic data of the potassium salt **7** suggest an unexpectedly high molecular symmetry, but unfortunately, a single-crystal structural analysis could not be performed to give details about the coordination mode of the two Cp^−^ ligands. Such information would be of particular interest to shed further light on the chemistry of the related trioxidotechnetium, which still represents a challenge in the field of organotechnetium chemistry.

There was (and is) a long-lasting debate about the existence and/or the stability of trioxidotechnetiumcyclopentadienyls. Given the importance of trioxorhenium(VII) compounds as catalysts or pre-catalysts [51] and the well-documented syntheses and structural characterization of [(η^5^-Cp*)ReO_3_] (Cp* = pentamethylcyclopentadienyl) and [(η^5^-CpMe_4_Et)ReO_3_] [52,53,54,55], the question arose as to the synthesis of related technetium compounds. Early attempts to follow the synthetic approach for the synthesis of [(η^5^-Cp*)ReO_3_], the oxidation of the corresponding tricarbonyl [(η^5^-Cp*)M(CO)_3_] with hydrogen peroxide, were less successful. In 1998, a paper was published describing the successful synthesis of an oxidotechnetium compound by this route. However, the isolated yellow needles were not assigned the expected (rhenium analogous) monomeric structure [(η^5^-Cp*)TcO_3_] (**8**), but the polymeric structure [Tc_2_O_3_(Cp*)]_n_ (**9**), with an extremely short Tc–Tc distance [56]. Subsequent re-evaluations of the described crystallographic and some spectroscopic results gave rise to doubts about this polymeric structure, and the possibility of the formation of a product with terminal oxido ligands (as compound **8**) was seriously discussed [55,57]. The question of the potential stability of [(η^5^-Cp*)TcO_3_] and [Tc_2_O_3_(Cp*)]_n_, including fluorinated species, has been evaluated by computational methods, concluding that both types of compounds should be thermodynamically stable [58,59,60]. Thus, more synthetic efforts seem to be necessary to find a suitable access to [(Cp)TcO_3_] species, also bearing in mind that a number of other stable trioxidotechnetium(VII) species have been isolated, including the organometallic [CH_3_TcO_3_] or ‘Cp^−^ mimics’ such as pyrazolylborates ({HB(pyrazolyl)_3_}^−^) and the ‘Kläui tripodal ligand’ {(C_5_H_5_)Co[(CH_3_O)_2_PO]_3_}^−^ [57,61,62].

## 3. Carbonyl Complexes

**Scheme 2:**
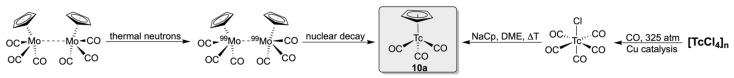
Early syntheses of [(Cp)Tc(CO)_3_] [63,66].

An extraordinary route was used for the synthesis of the first representative of the big family of (η^5^-cyclopentadienyl)tricarbonyltechnetium(I) compounds: the irradiation of the [(Cp)Mo(CO)_3_]_2_ dimer with thermal neutrons [63]. This results in a neutron capture of ^98^Mo and formation of the ^99^Mo (Scheme 2). The product is a β^−^-emitter with a half-life of 66 h and disintegrates with formation of ^99^Tc via the γ-emitting nuclear isomer ^99m^Tc, the radiation of which can readily be detected and distinguished from the β^−^ radiation of ^99^Mo. Thus, the volatile product [(Cp)Tc(CO)_3_] (**10a**) can be followed during several separation operations and assigned on the basis of its physical properties. In a similar way, another fundamental organotechnetium compound, the cationic [Tc(benzene)_2_]^+^, was prepared at tracer level before larger amounts became accessible [64].

Even when such syntheses of organotechnetium compounds are unusual and do not yield weighable amounts of the products, they are of general importance, since they give evidence that at least considerable amounts of the organometallic molybdenum starting materials resist the irradiation with neutrons and also recoil effects during the Mo/Tc element conversion do not destroy the organic skeletons significantly or at least allow re-formation of the stable molecules. A later, more detailed, mechanistic study indeed confirmed that the destruction of the organic fragments plays only a minor role [65]. Macroscopic amounts of compound **10** were first prepared in 1961 in good yields from [Tc(CO)_5_Cl] [66]. [Tc(CO)_5_Cl] proved to be an excellent starting material for the synthesis of several [Tc(Cp^R^)(CO)_3_] complexes and dominated as such the first decades of the Cp^−^ chemistry of technetium. A major drawback of this approach, however, is the need for autoclave reactions for the synthesis of the precursor molecules, which represent serious limitations with respect to the existing radiation protection rules in many laboratories. Thus, the development of normal-pressure syntheses for technetium carbonyl precursors such as (NEt_4_)_2_[Tc(CO)_3_Cl_3_] or (NBu_4_)[(CO)_3_Tc(µ-Cl)_3_Tc(CO)_3_] from pertechnetate represented an immense progress for the synthesis of tricarbonyltechnetium complexes and also allowed ready access to a cornucopia of cyclopentadienyl complexes [67,68,69,70]. In particular, the possibility to transfer such a synthetic approach to the metastable nuclide ^99m^Tc opened the door to the development of an extended aqueous tricarbonyltechnetium(I) chemistry with a high impact on the related radiopharmaceutical chemistry [12,71]. A short summary of the related research is given in Section 6.

**Scheme 3:**
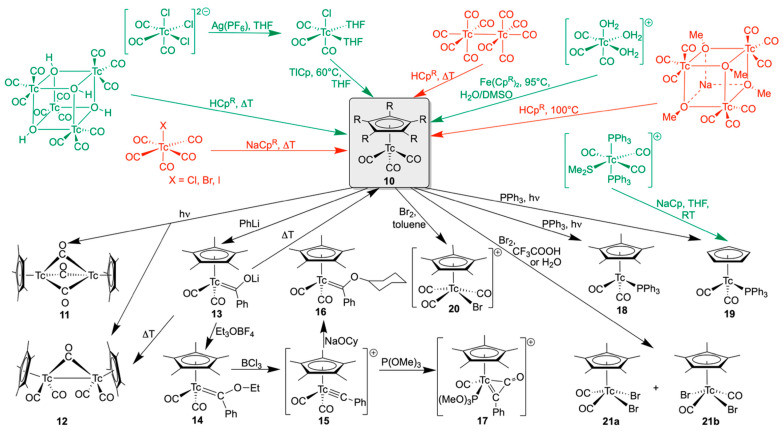
Syntheses and reactions of [(Cp^R^)Tc(CO)_3_] complexes. Starting materials, which are accessible without autoclave reactions, are drawn in green, while those that require high-pressure reactions are shown in red.

Scheme 3 contains a summary of the main synthetic approaches to [Tc(Cp^R^)(CO)_3_] compounds and some of their reactions. ‘Classical’, autoclave-based starting materials such as [Tc_2_(CO)_10_] and [Tc(CO)_5_X] (X = Cl, Br, I) (in red color) are complemented by carbonyltechnetium compounds, which can be synthesized by ‘normal pressure reactions’ (in green color): (NEt_4_)_2_[Tc(CO)_3_Cl_3_] [67,68], its dimeric analog (NBu_4_)[(CO)_3_Tc(µ-Cl)_3_Tc(CO)_3_] [69,70], and their solvolysis products, also including trimeric or tetrameric cluster compounds [72,73]. Reactions with common Cp^R−^ sources such as the corresponding alkali metal salts or the neat parent hydrocarbons (HCp^R^) are usually conducted in inert solvents and give good yields. Frequently, the purification of the products by sublimation, extraction, or chromatographic methods was required. Such procedures are sometimes challenging with respect to the radioactivity of technetium. However, generally, the [(Cp^R^)Tc(CO)_3_] products could be obtained in good yields. Thus, a remarkable collection of such compounds with a large variety of organic residues has been isolated as starting materials for ongoing studies.

**Figure 2:**
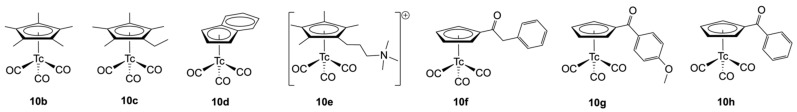
[(Cp^R^)Tc(CO)_3_] complexes characterized by single-crystal X-ray diffraction [74,75,76,77].

Figure 2 depicts the structures of some representatives, which have been studied by X-ray diffraction. It is evident that not only alkyl-substituted Cp^−^ ligands (**10b**, **10c**) have been prepared but also compounds with reactive residues (**10f**, **10g**) and quaternized amino groups (**10e**) in the Cp^−^ rings, or those with fused rings (**10d**), have received consideration. This structural manifold recommends such products for further substitutions towards potential applications, e.g., as potential labeling molecules in nuclear medicine.

Further options for modifications include reactions on the carbonyl co-ligands, their substitution, or the oxidation of the technetium(I) species. Indeed, such reactions have already been reported shortly after the first synthesis of the parent compound [(Cp)Tc(CO)_3_] (**10a**). Treatment of **10a** with benzoyl chloride and AlCl_3_ in CS_2_ resulted in acylation of the coordinated cyclopentadienyl ring (**10h**) [78].

The irradiation of compound **10b** with UV light results in cleavage of Tc-CO bonds and the formation of the dimeric compounds [(Cp*)Tc(µ-CO)_3_Tc(Cp*)] (**11**) and [(Cp*)(CO)_2_Tc(µ-CO)Tc(CO)_2_(Cp*)] (**12**). A structure determination on compound **11** reveals a Tc–Tc distance of 2.413 Å, which is discussed as a triple bond, while a Tc–Tc single bond is concluded from spectroscopic data for the dimer **12** [79]. Rhenium analogs of both complexes are formed in a similar reaction [80]. The heating of compound **12** in a vacuum leads to decomposition and the formation of a mixture of **10b** and **11**.

Nucleophilic attacks on the carbonyl ligands of **10b** follow common reaction patterns observed for other metal carbonyls. Thus, the acyl complex Li[(Cp*)Tc(CO)_2_{C(O)Ph}] (**13**) is formed upon reaction with LiPh [81]. The addition of (Et_3_O)BF_4_ converts **13** into the corresponding Fischer-type carbene [(Cp*)Tc(CO)_2_{C(OEt)Ph}] (**14**), which is a crystalline, air-stable, yellow compound and can be sublimed at 100 °C without decomposition. Treatment with BCl_3_ in pentane at low temperatures converts the carbene into the cationic carbyne [(Cp*)Tc(CO)_2_(CPh)](BCl_4_) (**15**), which shows a remarkable thermal stability. Subsequent conversion into a mixture of compounds **10b** and **12** proceeds at temperatures > 120 °C. The carbyne carbon atom in compound **15** is more electrophilic than the central Tc or the CO co-ligands, allowing nucleophilic reactions. A corresponding reaction with sodium cyclohexanolate yields yellow crystals of the alkoxycarbene [(Cp*)Tc(CO)_2_(PhCOCy)] (**16**), while a green η^2^-bonded ketenyl compound [(Cp*)Tc(CO){P(OMe)_3_}(PhC-C=O)]^+^ (**17**) is described as the product of a carbyne–carbonyl coupling under the influence of trimethylphosphite. P(OMe)_3_ is bonded as a co-ligand in the resulting technetium complex **17**. Contrarily, a similar reaction with the analogous rhenium compound [(Cp*)Re(CO)_2_(CPh)]Cl results in a clean addition of P(OMe)_3_ to the carbyne and the formation of the red ylide [(Cp*)Re(CO)_2_{PhCP(OMe)_3_})]Cl [81].

While the syntheses of [(Cp*)Tc(CO)_2_X] dicarbonyl compounds succeed via nucleophilic attacks on CO carbon atoms and subsequent conversions [81], direct thermal substitution reactions of CO ligands have not been reported for technetium(I). Having in mind the observed carbonyl abstraction during the irradiation of [(Cp*)Tc(CO)_3_] with UV light and the ready formation of the dimeric compounds **11** and **12**, such photochemical reactions have been found to be a feasible alternative approach for carbonyl substitution. This has been shown by the synthesis and structural characterization of [(Cp*)Tc(CO)_2_(PPh_3_)] (**18**) and [(Cp)Tc(CO)_2_(PPh_3_)] (**19**) [82]. It is not unexpected that the yields obtained during such photochemical procedures are often limited due to low selectivity or side reactions, and chromatographic methods must be applied to obtain pure products. An alternative procedure for the synthesis of the Cp^−^ derivative **19** is a ligand exchange procedure starting from *mer*-[Tc(CO)_3_(PPh_3_)_2_(SMe_2_)]^+^. The labile dimethylsulfide ligand in this compound is readily replaced under mild conditions by numerous ligands, and a corresponding reaction with NaCp gives a pure product in a practically quantitative yield at room temperature [83]. The convenience of this relatively new approach to organotechnetium compounds also recommends *mer*-[Tc(CO)_3_(PPh_3_)_2_(SMe_2_)] as a starting material for the synthesis of other (substituted) Cp^−^ derivatives.

The oxidation of [(Cp^R^)Tc(CO)_3_] complexes by elemental bromine results in oxidation of the metal ions and the formation of corresponding technetium(III) complexes. Initial experiments of such reactions ([(Cp*)Tc(CO)_3_] + Br_2_ in trifluoroacetic acid) gave a relatively clear picture of the formed products: *cis*- (**21a**) and *trans*-[(Cp*)Tc(CO)_2_Br_2_] (**21b**). Both products could be isolated by chromatographic methods and structurally assigned by their characteristic IR frequencies [75]. A more detailed study of this oxidation revealed a more elaborate pattern. The oxidation of **10b** with Br_2_ in CH_2_Cl_2_ is not exclusively restricted to the metal, but also concerns the Cp*^−^ ring system, which results in bond cleavage and the parallel formation of the dimeric anion [(CO)_3_Tc(µ-Br)_3_{Tc(CO)_3_]^−^ and [(Cp*)Tc(CO)_3_Br]^+^ (**20**) [84]. The choice of the solvent is crucial for the preference of individual reactions and the isolation of defined products. Thus, it has been found that the reaction between equivalent amounts of **10b** and Br_2_ in cold toluene initially gives [(Cp*)Tc(CO)_3_Br]Br ([**20**]Br) in almost quantitative yield [84]. This product, however, is photochemically and thermally unstable and rapidly undergoes bromide-dependent redox reactions, as has been studied for the related rhenium compounds. The driving force is the reduction of the metal ions by Br^−^ and the formation of [(CO)_3_M(µ-Br)_3_{M(CO)_3_]^−^ anions, which contain the metals in the oxidation state ‘+1’ [84]. The reaction between [(Cp*)Tc(CO)_3_] (**10b**) and Br_2_ in water gives the two [(Cp*)Tc(CO)_2_Br_2_] isomers **21a** and **21b**, which have previously been described as products for similar reactions in trifluoroacetic acid [75].

The volatility of [(Cp)Tc(CO)_3_] made this compound one of the candidates (besides [Tc_2_(CO)_10_], [TcX(CO)_5_], and [TcX(CO)_3_]_4_ (X = halide)) for vapor deposition experiments with the goal to produce defined technetium layers on metal surfaces [85,86]. The Cp^−^ complex was found to be suitable as a potential candidate for such potential applications.

**Scheme 4:**
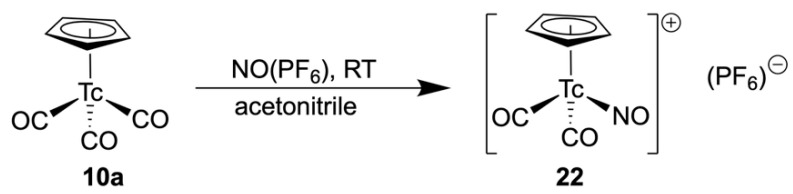
Synthesis of [(Cp*)Tc(NO)(CO)_2_](PF_6_) (**22**).

Although the {Tc^I^(CO)_2_(NO)}^2+^ core is well known and the approach to several complexes with various ligand systems is established [87,88], the related chemistry with cyclopentadienyl compounds is scarcely explored. This is surprising, since the only report about such a compound, [(Cp*)Tc(NO)(CO)_2_](PF_6_) (**22**), describes a versatile access to such compounds, namely the treatment of [(Cp*)Tc(CO)_3_] with NO(PF_6_) in acetonitrile. It gives compound **22** in a clean reaction and with high yields (Scheme 4). IR spectroscopy clearly shows the parallel presence of CO and NO^+^ ligands with their typical bands at 2035 and 1745 cm^−1^ [75].

More cyclopentadienyl complexes of technetium with accompanying nitrosyl ligands are described in the following section, which summarizes the syntheses and reactions of compounds with a central {(Cp^R^)Tc(NO)(PPh_3_)}^+^ core.

## 4. Nitrosyl Complexes

**Scheme 5:**
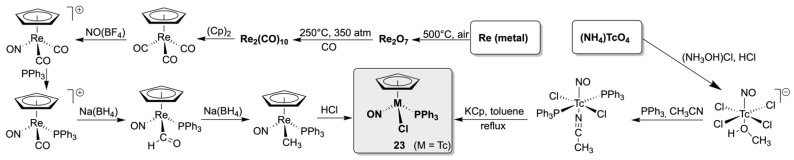
Comparison of the synthetic approaches to [(Cp)M(NO)(PPh_3_)Cl] complexes established for M = Re and Tc.

There has been extensive chemistry research into rhenium compounds containing a central {(Cp^R^)Re(NO)(PPh_3_)}^+^ core. They were comprehensively studied during the recent decades by John Gladysz and his co-workers. The results are summarized in more than a hundred research papers, of which only a few are cited here [89,90,91,92,93,94,95,96,97,98,99,100,101,102,103,104,105,106,107,108,109,110,111,112,113,114,115,116,117], and a vast number of more than 200 crystal structures of such compounds can be found in the Cambridge structural database [118]. Much less is known about the related technetium chemistry. This is mainly due to the synthetic approach to the key compound of this class, [(Cp)Re(NO)(PPh_3_)Cl]. It is commonly synthesized in a multi-step reaction scheme starting from decacarbonyldirhenium(0). The corresponding reaction sequence is shown in Scheme 5, and it is clear that the number of reaction steps and, in particular, the use of the volatile decacarbonyl, whose synthesis requires a high-pressure reaction, is not particularly suitable for use with the radioactive element technetium. The search for a synthesis method compatible with the use of radioactive materials was therefore the crucial point for the establishment of the corresponding technetium chemistry. It was found with a three-step route, which is depicted in Scheme 5. The reaction sequence starts directly from pertechnetate and allows a convenient synthesis of [(Cp)Tc(NO)(PPh_3_)Cl] (**23**), which works entirely at normal pressure and delivers the product as red crystals in a satisfactory overall yield [119]. Compound **23** is stable in air and readily soluble in organic solvents such as CH_2_Cl_2_, THF, or acetonitrile, making it a perfect starting material for further reactions.

**Scheme 6:**
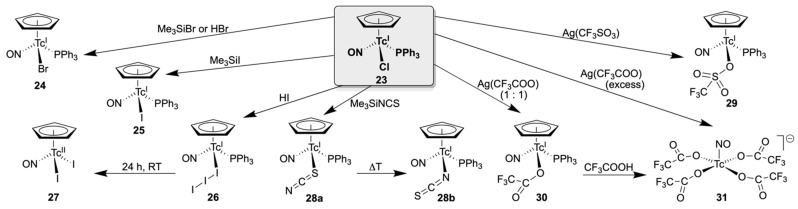
Ligand exchange of [(Cp)Tc(NO)(PPh_3_)Cl] with anionic ligands.

In particular, the chlorido ligand in compound **23** is relatively labile and can be replaced by simple metathesis reactions. Such procedures give easy access to structurally similar species with other anionic ligands such as halides, pseudohalides, carboxylates, or sulfonates (Scheme 6) [120]. Usually, the ligand exchange is supported by the addition of chloride scavengers such as silver ions or trimethylsilyl compounds. They can be conducted in organic solvents, and the products are commonly formed as colored, crystalline solids. Reasonable reaction times are achieved by heating. Straightforward chloride/halide or chloride/pseudohalide exchanges are observed with Me_3_SiBr, Me_3_SiI and Me_3_SiNCS [119,120], during which [(Cp)Tc(NO)(PPh_3_)Br] (**24**), [(Cp)Tc(NO)(PPh_3_)I] (**25**), and [(Cp)Tc(NO)(PPh_3_)(κS-SCN)] (**28a**) are formed in good yields. The ν_NO_ IR frequencies of the technetium(I) complexes are observed between 1670 and 1690 cm^−1^, which reflects a considerable backdonation from the electron-rich metal centers into ligand orbitals. The nitrosyl ligands of the isolated [(Cp)Tc(NO)(PPh_3_)X] complexes are linearly coordinated as in all other studied nitrosyl complexes of technetium [118,121] and can be understood as NO^+^ units.

The bromido product **24** is also obtained during a reaction of **23** with hydrobromic acid [119], while a similar treatment of **23** with HI gives the triiodido complex [(Cp)Tc(NO)(PPh_3_)(I_3_)] (**26**) instead. The formation of the triiodide in the latter reaction is described as an (possibly metal-mediated) oxidation of iodide directly in the reaction mixture, since it was also observed when previously carefully purified HI was used. Interestingly, the formed triiodido complex **26** also shows an unexpected reactivity. While the solid compound is stable, solutions of **26** in CH_2_Cl_2_ gradually change their color from dark red to bright green at room temperature. The observed reaction can be followed spectroscopically, indicating that the starting compound is completely converted within 24 h. EPR spectroscopy and X-ray diffraction proved that the product is a technetium(II) compound of the composition [(Cp)Tc(NO)(I)_2_] (**27**), obviously as the result of an internal redox reaction due to the decomposition of triiodide. It should be noted that the oxidation of the metal ion resulted in the degradation of the central {(Cp)Tc(NO)(PPh_3_)}^+^ core. An internal conversion is also observed for the isolated thiocyanato complex **28a**. The initially formed, *S*-coordinated compound is indeed the first thiocyanato complex of technetium, while all other structurally characterized compounds with this ligand are *N*-coordinated isothiocyanato derivatives [122,123,124,125,126,127,128,129,130,131]. It represents, however, a ‘kinetic product’ and prolonged heating of solutions of **28a** gave the isomeric compound [(Cp)Tc(NO)(PPh_3_)(κN-NCS)] (**28b**). The higher stability of **28b** over **28a** was confirmed by DFT calculations, which gave an energetic preference of this isomer by 22 kJ/mol [120]. The use of corresponding silver salts allowed the synthesis of {(Cp)Tc(NO)(PPh_3_)}^+^ complexes with oxygen donors such as trifluoromethylsulfonate (**29**) and trifluoroacetate (**30**). For the synthesis of **30**, it was important to use only one equivalent of Ag(CF_3_COO), since the organometallic skeleton of the starting material **23** is destroyed by an excess of the reagent, and the technetium(II) anion [Tc(NO)(CF_3_COO)_4_]^−^ (**31**) is formed. Compound **31** can also be obtained by the heating of **30** in neat CF_3_COOH [120].

**Scheme 7:**
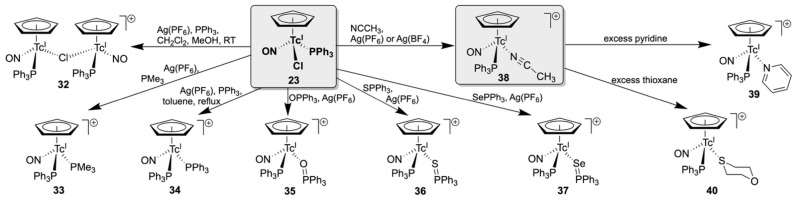
Ligand exchange of [(Cp)Tc(NO)(PPh_3_)Cl] complexes with neutral ligands.

Expectedly, cationic cyclopentadienyl complexes of technetium(I) are the products of reactions of complex **23** with neutral ligands or by removal of the chlorido ligand with silver ions. A summary of the related structural chemistry is depicted in Scheme 7. Treatment of compound **23** with Ag(PF_6_) or Ag(BF_4_) in non-coordinating solvents such as CH_2_Cl_2_ or toluene resulted in the precipitation of AgCl and the formation of the chlorido-bridged compound [{(Cp)Tc(NO)(PPh_3_)}_2_(µ-Cl)] (**32**)]. It has been found that this dimer is a kind of ‘kinetic product’, that stabilizes the {(Cp)Tc(NO)(PPh_3_)}^+^ core in cases where no suitable ligand for the immediate replacement of Cl^−^ is available (e.g., during reactions with alkynes) or such reactions are kinetically unfavorable [132]. A good example for the latter case is given with reactions of the starting complex **23** with mixtures of Ag(PF_6_) and triphenylphosphine. In boiling CH_2_Cl_2_, they result in the formation of the red dimer **32**, while similar reactions under harsher conditions (e.g., in boiling toluene) give the orange-yellow bis(triphenylphosphine) complex **33** [132]. In the coordinating solvent acetonitrile, the corresponding cationic complex [(Cp)Tc(NO)(PPh_3_)(NCCH_3_)]^+^ (**38**) is obtained.

Similarly, the presence of suitable incoming ligands such as PMe_3_, or triphenylphosphine chalcogenides result in the formation of the corresponding complex cations [(Cp)Tc(NO)(PPh_3_)(PMe_3_)]^+^ (**33**), [(Cp)Tc(NO)(PPh_3_)(OPPh_3_)]^+^ (**35**), [(Cp)Tc(NO)(PPh_3_)(SPPh_3_)]^+^ (**36**) or [(Cp)Tc(NO)(PPh_3_)(SePPh_3_)]^+^ (**37**) [132,133]. The availability of structural, spectroscopic, and computational data of structurally strongly related compounds such as the PMe_3_/PPh_3_ derivatives **33** and **34** or the triphenylphosphine chalcogenide complexes **35**, **35,** and **37** allows conclusions to be drawn about the observed reaction pattern and the binding situation in the compounds. Thus, clear differences between the electron distributions in the E=PPh_3_ complexes have been found, indicating the presence of a O=P double bond in the triphenylphosphine oxide complex **35**, while in the sulfide and selenide analogs the P–S and P–Se bond orders are markedly decreased (interestingly with the highest extension found for the sulfide) [133]. Steric effects due to the ‘kinetic intermediate’ **32** and/or the higher basicity of PMe_3_ compared with PPh_3_ explain the ready formation of the PMe_3_ complex **33**, while more drastic conditions were required for the corresponding PPh_3_ compound **34** [132].

Kinetic effects are also discussed for the observed inertness of the acetonitrile complex **38**. Experiments concerning its suitability as a (chloride-free) precursor for further ligand exchange reactions confirmed previous results found for acetonitrile complexes with carbonyl co-ligands [134,135,136]; although the Tc-N bonds should be relatively weak with respect to their bond lengths, they are tightly bonded and can only be replaced by ligands with strong donor atoms or under harsh conditions. Attempted reactions with THF or DMSO, but also with a series of alkynes, failed, and notable ligand exchange products could only be obtained for the pyridine complex [(Cp)Tc(NO)(PPh_3_)(py)]^+^ (**39**) and the *S*-bonded thioxane derivative [(Cp)Tc(NO)(PPh_3_)(κS-thioxane)]^+^ (**40**). The kinetically inhibited ligand exchange behavior of the acetonitrile compound **38** was studied by ^15^N NMR experiments using ^15^N-enriched acetonitrile. Slow exchange rates between bound NCCH_3_ ligands and free NCCH_3_ were confirmed at room temperature [133]. It should additionally be noted that a (likely metal-mediated) decomposition of PF_6_^−^ limits the thermal stability of the compounds in solution, while BF_4_^−^ salts are more robust [133].

**Scheme 8:**
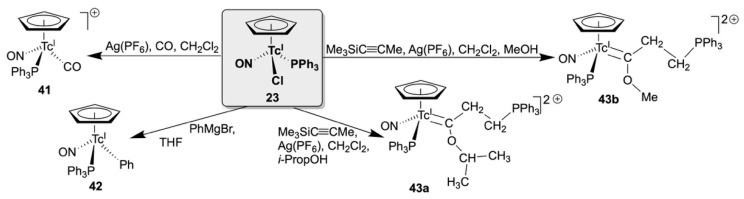
Substitution products of [(Cp)Tc(NO)(PPh_3_)Cl] containing a second organometallic unit.

Organometallic derivatives with the {(Cp)Tc(NO)(PPh_3_)}^+^ core can be obtained by the established routes, starting from the common precursor **23** (see Scheme 8). Bubbling CO gas through a solution of **23** containing one equivalent of Ag(BF_4_) yields the BF_4_^−^ salt of the carbonyl complex [(Cp)Tc(NO)(PPh_3_)(CO)]^+^ (**41**) as a red, crystalline substance. The phenyl derivative [(Cp)Tc(NO)(PPh_3_)(Ph)] (**42**) is obtained by a reaction of **23** with phenyl magnesium bromide [119]. Both compounds are air-stable and are potential starting compounds for further substitutions.

A more complex reaction is observed between complex **23**, Me_3_SiC≡CMe and Ag(PF_6_) in a CH_2_Cl_2_/ROH mixture, with the formation of ‘Fischer-type’ carbenes of the compositions [(Cp)Tc(NO)(PPh_3_){C(OR)(CH_2_CH_2_PPh_3_)}]^2+^ (**43a**: R = Me; **43b**: R = *i*-Prop) [132]. A complex reaction sequence is discussed for its formation, involving the intermediate coordination of an acetylido ligand. The source of the residue OR being the added alcohol is confirmed by changing this component. Similar alkenylphosphonio complexes have been obtained for ruthenium, resulting from reactions of vinylidene complexes with PPh_3_ [137,138].

**Scheme 9:**
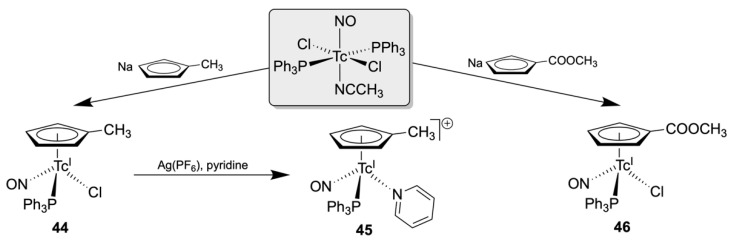
Synthesis and reactions of [(Cp^R^)Tc(NO)(PPh_3_)(L)] complexes (R = Me, COOMe) [133].

Only a few technetiumnitrosyl complexes with substituted Cp^R^ rings have hitherto been prepared. Due to the stability of their core structure, such compounds are of particular interest as suitable candidates for the synthesis of bioconjugates in a radiopharmaceutical context. They are shown in Scheme 9. While the Cp^Me^ derivatives **44** and **45** have been prepared as a ‘proof of principle’ for the inclusion of substituted cyclopentadienyls into the related core chemistry, the ester-substituted compound **46** allows further modifications of the basic structure of such complexes by simple and established standard procedures for bioconjugation [133].

With the synthesis of such chemically modifiable derivatives, together with the possibilities given by the chloride-exchange summarized vide supra, such nitrosyls might represent the foundation of novel chemical solutions in the search for further technetium-based radiopharmaceuticals. A general approach to a suitable ^99m^Tc nitrosyl starting material, [^99m^Tc(NO)Cl_4_]^−^, was developed some decades ago [139], but hitherto, no attempts have been undertaken to apply it for cyclopentadienyl-based solutions. It should be mentioned that the extremely low, nanomolar technetium concentrations in such solutions allow synthetic approaches to ^99m^Tc organotechnetium compounds from a larger variety of starting materials than discussed above, and even reactions in aqueous solutions become possible. A short summary of this point will be given in Section 6.

## 5. ^99^Tc NMR Spectroscopy on Cyclopentadienyl Complexes

Most of the cyclopentadienyl compounds of technetium are diamagnetic, and NMR spectroscopy is an important method for their characterization. As has already been mentioned in Section 2, sometimes couplings with the large quadrupole moment of ^99^Tc cause problems with considerable line broadenings in the spectra of nuclei that are directly bonded to technetium. Such effects are dependent on the local symmetry in the individual compounds and have been found for ^1^H [38,43], ^31^P [41,42,43,140], and ^15^N spectra [133,140]. In extreme cases, they completely prevent the detection of such spectra in reasonable quality. Symmetry-dependent line broadening is also found in ^99^Tc NMR spectra, but does, in most cases, not hinder the detection and interpretation of the corresponding resonances.

The ^99^Tc nucleus has a high molar receptivity (about 0.3 compared to ^1^H), which makes it one of the most receptive NMR nuclei and allows for the detection of reasonable signals even with relatively small amounts of the compounds. (NH_4_)TcO_4_ is used as a reference and the chemical shifts observed in the ^99^Tc NMR spectra span over a range of several thousand ppm [37,45,141]. Depending on the local symmetry around the central technetium atoms, line-widths between ν_1/2_ < 100 Hz (for highly symmetric compounds such as for the tetrahedral TcO_4_^−^ ion or the perfectly octahedral hexakis(isocyanide)technetium(I) cations [45]) and values larger than 20 kHz (e.g., for asymmetric mixed-isocyanide/halide complexes [142]) are observed. Nevertheless, ^99^Tc NMR is a powerful tool in synthetic technetium chemistry. The high receptivity of the nucleus and the large spectral range commonly allow the ready observation of the product resonances, but also the detection of intermediate species directly in reaction mixtures. This also applies to the diamagnetic cyclopentadienyl compounds reviewed in the present paper.

Table 1 summarizes the available ^99^Tc NMR data of Tc Cp complexes. They all belong to the above-discussed groups of Tc(I) carbonyl and nitrosyl compounds. It is evident that the chemical shift range of the measured signals is extremely large, particularly for the compounds with the {(Cp^R^)Tc(NO)(PPh_3_)}^+^ core (+242 ppm to −1753 ppm), while the signals of the tricarbonyl compounds (but also that of [(Cp)Tc(CO)_2_(PPh_3_)]) all appear between −2410 and −2502 ppm. Such high field values observed for the signals of the latter compounds are obviously the result of an efficient shielding of the ^99^Tc nucleus by the coordination of carbonyl ligands. Such a conclusion is supported by the appearance of the resonance of the nitrosyl/carbonyl species **41** at −1753 ppm, which is the lowest chemical shift hitherto observed for a compound of the [(Cp^R^)Tc(NO)(PPh_3_)(L)]^0,+^ family.

**Figure 3:**
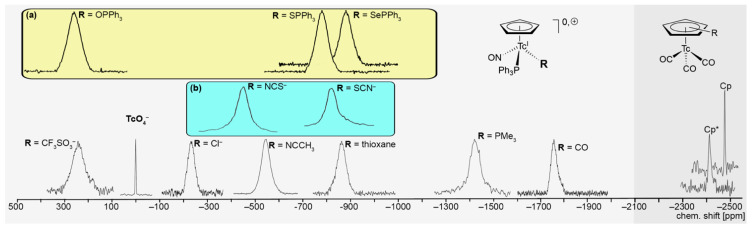
Typical ^99^Tc NMR spectra of some cyclopentadienyl complexes of Tc(I), illustrating the large differences in their chemical shifts and line-widths. The spectra in the colored boxes show (**a**) the spectra of the strongly related triphenylphosphine chalcogenide complexes **35**, **36**, and **37**, and (**b**) the spectra of the two isomers **28a** and **28b**. The area with the dark gray background indicate the range, where the signals of tricarbonyl complexes appear.

Figure 3 illustrates the comparison of some typical ^99^Tc NMR spectra of the compounds covered by this review, together with that of the reference compound (NH_4_)TcO_4_. At first glance, the different widths of the signals become evident. The line widths provide some information about the symmetry of the coordination sphere around the Tc(I) ion. Thus, an extremely narrow line is observed for the perfectly tetrahedral pertechnetate ion, while the coordination spheres of the ‘pseudo octahedral’ Cp^−^ complexes are inherently distorted by the occupation of three facial coordination positions by the π-bonded Cp^R^ ring systems. Likewise, the larger line widths observed for the [(Cp^R^)Tc(NO)(PPh_3_)(L)]^0,+^ compounds (typical line widths between 3000 and 7000 Hz) can be understood by a further increase in the asymmetry of the coordination sphere compared with the tricarbonyl complexes having three equal CO ligands, where line widths between 300 and 1200 Hz are observed. It should be noted that such line width considerations are more or less qualitative, and more parametrization is required to derive a physically exact instrument for reliable prediction of such parameters.

Another obvious feature is the appearance of clearly different expectation ranges for the nitrosyl and the carbonyl complexes and the large chemical shift differences within the studied nitrosyl compounds. Although the members of the [(Cp^R^)Tc(NO)(PPh_3_)(L)]^0,+^ product family are only distinguished by one single ligand, their signals appear over a chemical shift range of approximately 2000 ppm. This finding indicates a strong dependence of the observed ^99^Tc chemical shifts on the nature of the coordinated ligands. It readily allows conclusions about the presence, appearance, or disappearance of individual species in corresponding reaction mixtures by their ^99^Tc NMR monitoring. A nice example has been presented by the isomerization of [(Cp)Tc(NO)(PPh_3_)(κS-SCN)] (**28a**) into [(Cp)Tc(NO)(PPh_3_)(κN-NCS)] (**28b**), the ^99^Tc NMR signals are distinguishable by almost 400 ppm [120].

Additionally, the positions of the ^99^Tc resonances can be used as a first indicator for differences in some bonding parameters within structurally related complex molecules as has been performed for the triphenylphosphine chalcogenide complexes [(Cp)Tc(NO)(PPh_3_)(OPPh_3_)]^+^ (**35**), [(Cp)Tc(NO)(PPh_3_)(SPPh_3_)]^+^ (**36**) and [(Cp)Tc(NO)(PPh_3_)(SePPh_3_)]^+^ (**37**). The chemical shift recorded for the phosphine oxide compound **35** appears approximately 1000 ppm downfield compared with the values of its sulfur and selenium analogs **36** and **37**. X-Ray structure determinations and DFT consideration of the bonding situations clearly support the NMR-suggested differences in the electronic structures of these compounds [133].

**Figure 4:**
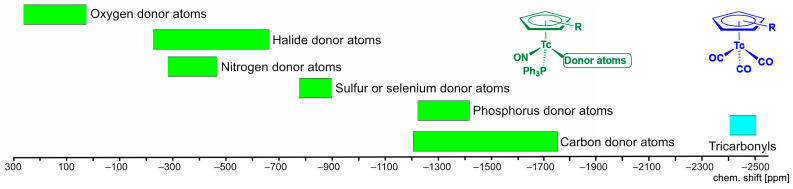
Typical expectation ranges for ^99^Tc chemical shifts in Tc(I) cyclopentadienyl complexes depending on their donor atoms.

The considerable number of related compounds with Cp^−^ ligands studied by ^99^Tc NMR spectroscopy allows us to derive typical expectation ranges for individual compounds depending on the individual donor atoms, as illustrated in Figure 4. The indicated ranges may overlap between different donor atoms and bonding situations, but provide an approximate estimation. More exact predictions might be possible with the aid of computational methods.

In summary, ^99^Tc NMR spectroscopy can be regarded as a powerful tool in synthetic technetium chemistry. This is due to the high receptivity of the ^99^Tc nucleus, combined with significant differences in the obtained spectral parameters (chemical shift, linewidth), which depend more or less systematically on the composition of the studied compounds. The method allows the estimation of diamagnetic technetium species in solution and supplies invaluable support for finding optimal synthesis pathways during the preparation of novel organotechnetium compounds.

## 6. ^99m^Tc Cyclopentadienyl Complexes in Nuclear Medical Research

At the beginning of this section, it should be noted that the first ever synthesized cyclopentadienyl compound of technetium, [(Cp)Tc(CO)_3_], was actually a ^99m^Tc complex. It was prepared by Baumgärtner and his colleagues in 1962 as the result of n,γ reactions performed on the dimeric molybdenum compound [(Cp)Mo(CO)_3_]_2_ [63] (see also Section 1). These experiments were clearly not conducted in the context of application-oriented nuclear medicine, but nevertheless, their results have considerable implications in this field. They demonstrate the extraordinary stability of the {(Cp^R^)Tc(CO)_3_} core structure, which even resists the energies related to nuclear conversions without considerable decomposition [65]. Another remarkable property of this structural motif is that the entire fragment can be considered as a bioisoster of a phenyl ring, showcasing the low influence of this central unit on the biological distribution patterns of labeled biomolecules. Consequently, several attempts were undertaken to find suitable approaches to ^99m^Tc compounds with (substituted) Cp^−^ ligands. An excellent review covering all such efforts and the successes achieved has been published recently by one of the protagonists in this field, Roger Alberto [143]. Chemists who are interested in the history and the current state of this branch of organotechnetium chemistry will find a wealth of information and valuable suggestions for their own projects there. It is therefore unnecessary to repeat all the details of the development of relevant Cp^−^ complexes of ^99m^Tc in the present work, allowing a highlighting of some main pathways and particularly important and/or noteworthy examples.

**Scheme 10:**
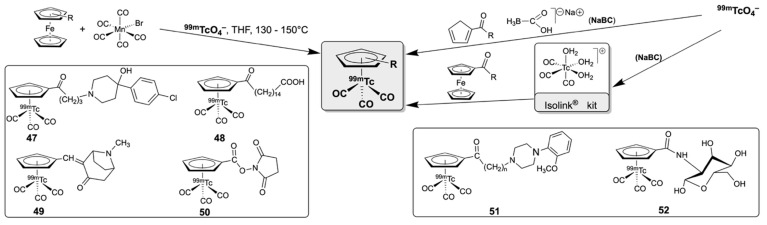
Selected synthetic approaches to [(Cp^R^)^99m^Tc(CO)_3_)] complexes and some of their products.

Bearing in mind that only extremely low concentrations of ^99m^Tc are available and required for the diagnostic applications in nuclear medicine, e.g., through commercial ^99^Mo/^99m^Tc generator systems (typically nanomolar), it becomes clear that corresponding syntheses are always conducted with a large excess of the non-technetium starting materials. Although this sounds disadvantageous at first glance, it allows unusual synthetic approaches such as ‘double ligand transfer’ reactions. This means that the components required for the target molecule (here, the carbonyl ligands and the Cp^R−^ ligands) are provided by different precursor molecules and are practically simultaneously transferred to the (reduced) ^99m^Tc ion. Such an approach is also one of the first that has been applied for the synthesis of Cp-based, potential radiodiagnostic technetium agents, and is shown in Scheme 10, together with other synthetic solutions developed more recently.

The first ‘double ligand transfer’ reactions involving ^99m^Tc cyclopentadienyls were conducted by Wenzel and co-workers in the early 1990s. Several (substituted) [(Cp^R^)^99m^Tc(CO)_3_] complexes were prepared from mixtures of ^99m^TcO_4_^−^, [Mn(CO)_5_Br] and substituted ferrocenes in THF/water or alcohol/water at temperatures between 130 and 150 °C [144]. The organic residues involved also cover medicinally relevant motifs such as haloperidol (**47**), fatty acids (**48**), and tropinone (**49**), but also cholesterol and glucosamines and a variety of other organic amines [144,145,146,147]. The first biological studies showed interesting distribution patterns for some of the products [145,146,147]. However, the use of organic solvents for such reactions, the high reaction temperatures, as well as the need for extensive purification operations were serious drawbacks on the way to clinical applications. A particular problem arises from the high reaction temperature, which restricts this method to application in robust organic molecules and made it unsuitable for labeling more sensitive biomolecules such as peptides and proteins. This issue was overcome a few years later by Katzenellenbogen and co-workers through developing a kind of post-labeling procedure. In the first step, a reactive succinimidyl ester functionalized Cp^−^ derivative (**50**) was prepared using the established high-temperature procedure (see Scheme 10). This compound can be purified and then used in a subsequent room-temperature reaction to couple sensitive molecules such as peptides to the coordinated Cp^−^ ring as has been demonstrated by the synthesis and biological evaluation of a corresponding octreotide derivative [148,149]. It should be mentioned that [Cr(CO)_6_] was used as carbonyl source in these experiments and such protocols also work with ^99^Tc, underlining the general character of the applied ‘double ligand transfer’ strategy.

The development of a normal pressure, aqueous synthesis of technetium carbonyls by Alberto and his co-workers represented a significant breakthrough and the beginning of a new era in bioorganotechnetium chemistry [67,71]. This had naturally also a major impact on the related cyclopentadienyl chemistry. Sodium borane carbonate, Na_2_[H_3_BCOO] was found to be a suitable CO source and reductant for ^99m^TcO_4_^−^, which forms the aqua complex [^99m^Tc(CO)_3_(OH_2_)_3_]^+^ under mild conditions, directly from commercially available ^99^Mo/^99m^Tc generator eluates (Scheme 10). The synthetic approach was transferred into a commercial kit called *Isolink^®^* [71] (for details about this kit see ref. [12]). The development of a facile synthesis for the reactive starting material [^99m^Tc(CO)_3_(OH_2_)_3_]^+^ and particularly the availability of a pre-manufactured kit multiplied the number of researchers, who were able to contribute with their ideas to the development of technetium carbonyl chemistry. This explicitly includes the large number of creative chemists working in nuclear medicine clinics, where equipment for pressure reactions or for working with ^99^Tc compounds is normally not available.

The first Cp^−^ complexes following the then newly established one-pot procedure were prepared from a number of α-carbonyl-substituted cyclopentadienes, which are easily accessed through standard organic chemistry derivatization strategies. This approach delivered the corresponding cyclopentadienyls in reasonable yields and purity, including a group of serotonergic receptor-binding molecules (**51**), shown in Scheme 10 [76,150,151]. The combination of the developed direct labeling of carbonyls with the use of (substituted) ferrocenes, as demonstrated by the Alberto group in 2004 [77], enlarged the number of available synthons significantly and has been adopted by others, e.g., for the synthesis of several glucosamine derivatives such as compound **52** in Scheme 10 [152,153,154]. More examples for the syntheses of [(Cp^R^)^99m^Tc(CO)_3_] derivatives following the labeling procedures outlined in Scheme 10 are compiled in Ref. [143] and can be found in more original communications [155,156,157,158,159,160,161,162,163,164].

**Scheme 11:**
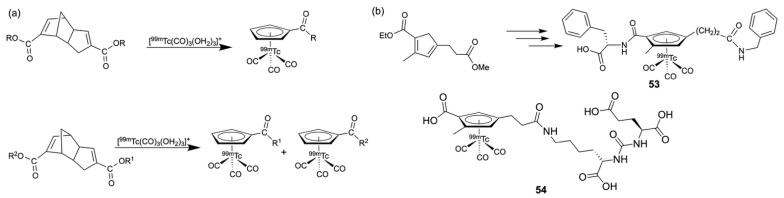
(**a**) Retro Diels–Alder reactions as synthetic approaches to [(Cp^R^)^99m^Tc(CO)_3_)] complexes and (**b**) bifunctional Cp^−^ ligands and their [(Cp^R^)^99m^Tc(CO)_3_)] complexes.

With the availability of well-suited ^99m^Tc precursors, it became interesting to optimize the substitution on the Cp^−^ rings and to simultaneously explore novel precursor molecules. Some new approaches concern an unusual synthetic pathway to Cp^−^ complexes by a retro Diels–Alder reaction or attempts to include Cp^−^ rings with multifunctional organic residues, allowing either ‘double labeling’, increasing the target binding rates, or ‘bifunctional labeling’, addressing two biological targets at once. Both examples are summarized in Scheme 11.

One approach to new starting materials for Cp^−^ ligands was found with (substituted) Thiele’s acid derivatives, which undergo a metal-mediated retro Diels–Alder reaction under the influence of [^99m^Tc(CO)_3_(OH_2_)_3_]^+^ and form the corresponding [(Cp^R^)^99m^Tc(CO)_3_)] complexes (Scheme 11a) [165,166,167,168,169]. This type of reaction allows the synthesis of several new derivatives by a convenient synthesis starting from appropriately derivatized Thiele’s acid. The reactions can be conducted in solution, but a solid-state version has also been developed, which allows the labeling to be performed on a resin [166]. The use of non-symmetric derivatives of Thiele’s acid results in the parallel formation of two differently substituted products, which, under special circumstances, is desired from the clinical side, e.g., when two biological target structures need to be monitored in parallelly [167].

The attachment of two different targeting groups to the Cp^−^ ring has also been demonstrated by the synthesis of a cyclopentadiene having two different carboxylic ester groups, which can be used to attach two different biologically active residues independently. An example is shown with the bis-substituted cyclopentadiene in Scheme 11b. The selectivity of the attempted substitutions was achieved by the introduction of a spacer between the ring and one of the carboxylic groups, which sufficiently changes the chemical reactivities of the two substituents [170,171,172]. Starting from this compound, the prototypical complex **53** with benzylamine and phenylalanine as biological model vectors could be synthesized [171]. A more practical example is shown with molecule **54**, where one of the reactive sites is attached to a prostate-specific membrane antigen (PSMA) ligand. It should be noted that the bis-substituted product can also be synthesized by slight modification of the experimental conditions, and both compounds show different affinities in cell binding assays [172].

To summarize, considerable efforts have been made in the ^99m^Tc-labeling chemistry using cyclopentadienyl units as anchors. The corresponding ‘organometallic procedures’ can nowadays be performed in water using standard ^99^Mo/^99m^Tc generator eluates in saline solutions. Several synthetic approaches to such compounds can be used to find the optimal conditions for the planned application, and bifunctional solutions are available to address specific diagnostic demands. Together with homologous, beta-emitting ^185/188^Re complexes, the ^99m^Tc compounds represent ‘matched pairs’ of diagnostic (^99m^Tc) and therapeutic (^185,188^Re) isostructural molecules to be applied in theranostic procedures.

## 7. Conclusions and Perspectives

The cyclopentadienyl chemistry of technetium is a growing field of research that has seen numerous new discoveries in recent years. Originally motivated by a fundamental interest in peculiar differences in the chemistry of the other group seven elements, manganese and rhenium, several novel aspects of the related structural chemistry have been explored. A recently developed class of compounds concerns stable nitrosyl complexes, whose rhenium analogs are already well-known. They show a well-defined substitution chemistry and have contributed fundamentally to a more systematic understanding of the spectral properties of technetium Cp^−^ complexes.

Starting from systematic studies, particularly on tricarbonyl complexes with the long-lived isotope ^99^Tc, the related chemistry has been successfully translated to the nanomolar concentration level of the γ-emitting, short-lived ^99m^Tc compounds. A wealth of research results has been produced towards the aim of the application of corresponding [(Cp^R^)^99m^Tc(CO)_3_] compounds in nuclear medical diagnostics.

The development of labeling techniques for complexes containing Cp^−^ ligands functionalized with biologically relevant molecules holds promise for further advances in the diagnosis of various diseases and the imaging of several target tissues, including the fields of brain imaging, myocardial imaging, and cancer imaging. A relatively novel, but important, field is the development of infection tracers. More details about the recent efforts to increase the efficiency of the biological targeting due to receptor-binding chemical solutions are found in the recent review about ^99m^Tc cytectrenes by Lengacher and Alberto [143].

The expected progress in the biological targeting of ^99m^Tc imaging agents will make them interesting for a combined use with related β^−^-emitting ^186,188^Re compounds as ‘matched pairs’ of isotopes in theranostic procedures.

## Figures and Tables

**Table 1:** ^99^Tc NMR data for cyclopentadienyl compounds of technetium.

Compound	δ [ppm]	ν_1/2_ [Hz]	Solvent	Ref.
[(Cp)Tc(NO)(PPh_3_)(CF_3_SO_3_)] (**29**)	242	7070	CDCl_3_	[120]
[(Cp)Tc(NO)(PPh_3_)(OPPh_3_)]^+^ (**35**)	241	6200	CD_2_Cl_2_	[133]
[(Cp)Tc(NO)(PPh_3_)(CF_3_COO)] (**30**)	19	4690	CDCl_3_	[120]
[{(Cp)Tc(NO)(PPh_3_)}_2_(µ-Cl)]^+^ (**32**)	−220	4150	CD_2_Cl_2_	[132]
[(Cp)Tc(NO)(PPh_3_)Cl] (**23**)	−231	7170	CDCl_3_	[119]
[(Cp^COOMe^)Tc(NO)(PPh_3_)Cl] (**46**)	−253	3350	CD_2_Cl_2_	[133]
[(Cp)Tc(NO)(PPh_3_)(py)]^+^ (**39**)	−285	4700	CDCl_3_	[133]
[(Cp^Me^)Tc(NO)(PPh_3_)Cl] (**44**)	−295	4750	CDCl_3_	[133]
[(Cp^Me^)Tc(NO)(PPh_3_)(py)]^+^ (**45**)	−300	4850	CDCl_3_	[133]
[(Cp)Tc(NO)(PPh_3_)Br] (**24**)	−359	6500	CDCl_3_	[119]
[(Cp)Tc(NO)(PPh_3_)(NCS)] (**28b**)	−453	6580	CDCl_3_	[120]
[(Cp)Tc(NO)(PPh_3_)(NCCH_3_)]^+^ (**38**)	−540	5900	CDCl_3_	[133]
[(Cp)Tc(NO)(PPh_3_)I] (**25**)	−668	4200	CDCl_3_	[120]
[(Cp)Tc(NO)(PPh_3_)(I_3_)] (**26**)	−679	6860	CDCl_3_	[120]
[(Cp)Tc(NO)(PPh_3_)(SPPh_3_]^+^ (**36**)	−783	5800	CD_2_Cl_2_	[133]
[(Cp)Tc(NO)(PPh_3_)(SCN)] (**28a**)	−820	6580	CDCl_3_	[120]
[(Cp)Tc(NO)(PPh_3_)(κ*S*-thioxane)]^+^ (**40**)	−860	5400	CDCl_3_	[133]
[(Cp)Tc(NO)(PPh_3_)(SePPh_3_)]^+^ (**37**)	−876	4850	CD_2_Cl_2_	[133]
[(Cp)Tc(NO)(PPh_3_)(Ph)] (**42**)	−1201	8820	CDCl_3_	[119]
[(Cp)Tc(NO)(PPh_3_)_2_]^+^ (**34**)	−1219	6600	CDCl_3_	[132]
[(Cp)Tc(NO)(PPh_3_){=C(O*i*-Prop)((CH_2_)_2_PPh_3_)}]^2+^ (**43a**)	−1370	4060	CDCl_3_	[132]
[(Cp)Tc(NO)(PPh_3_){=C(OMe)((CH_2_)_2_PPh_3_)}]^2+^ (**43b**)	−1371	3900	CDCl_3_	[132]
[(Cp)Tc(NO)(PPh_3_)(PMe_3_)]^+^ (**33**)	−1420	4100	CDCl_3_	[132]
[(Cp)Tc(NO)(PPh_3_)(CO)]^+^ (**41**)	−1753	3900	CDCl_3_	[119]
[(Cp*)Tc(CO)_3_)] (**10b**)	−2410	1130	CD_2_Cl_2_	This work ^(a)^
[(Cp)Tc(CO)_2_(PPh_3_)] (**19**)	−2420	2040	CD_2_Cl_2_	[43] ^(a)^
[(Cp)Tc(CO)_3_)] (**10a**)	−2477	330	CD_2_Cl_2_	This work ^(a)^
[(Cp^C(O)PhOMe^)Tc(CO)_3_]^+^ (**10g**)	−2478	^(b)^	CDCl_3_	[77]
[(Cp^C(O)CH2Ph^)Tc(CO)_3_]^+^ (**10f**)	−2502	740	MeOD	[76]

^(a)^ Somewhat different data have been published in earlier work [68,73]. ^(b)^ Not reported.

## Data Availability

No new data were created or analyzed in this study. Data sharing is not applicable to this article.
